# ROS Is Necessary for Dormancy Breaking in Fission Yeast Spores, While Its Downregulation by Sod1 Is Crucial for Later Events

**DOI:** 10.1111/gtc.70157

**Published:** 2026-11-03

**Authors:** Tatsuya Hasegawa, Masamitsu Sato

**Affiliations:** ^1^ Laboratory of Cytoskeletal Logistics, Department of Life Science and Medical Bioscience, Graduate School of Advanced Science and Engineering Waseda University Shinjuku‐ku Tokyo Japan; ^2^ Institute for Medical‐Oriented Structural Biology Waseda University Shinjuku‐ku Tokyo Japan

**Keywords:** dormancy, fission yeast, germination, reactive oxygen species (ROS), spore

## Abstract

Fission yeast spores adapt to nutrient deprivation by entering a dormant state. Upon exposure to nutrients, spores initiate germination, followed by proliferation. However, the molecular mechanisms that govern germination remain poorly understood. Here we demonstrate that an optimal level of reactive oxygen species (ROS) facilitates germination progression, with the level decreasing as germination proceeds. Observations using ROS fluorescent probes indicated that intracellular ROS was relatively high in dormant spores compared to germinating spores, as it decreased in response to germination cues. Nonetheless, artificially reducing ROS levels in dormant spores with a drug inhibited the initiation of germination, indicating that an adequate level of ROS is required to initiate germination. In later stages, however, a reduction in ROS is necessary because spores lacking the antioxidant enzyme Sod1 (copper‐zinc superoxide dismutase 1) fail to reduce ROS during these stages, hindering subsequent development. Thus, we conclude that intracellular ROS levels, which varied in accordance with the chronological sequence of spore germination, regulate the germination process.

## Graphical abstract

Dormant fission yeast spores germinate on exposure to nutrients. ROS promotes initiation of germination, whereas reduction of ROS is required in later stages of germination. Artificially lowering ROS at the initial stage inhibited germination, whereas elevating ROS later failed to promote it, indicating that optimal ROS levels are required for germination.

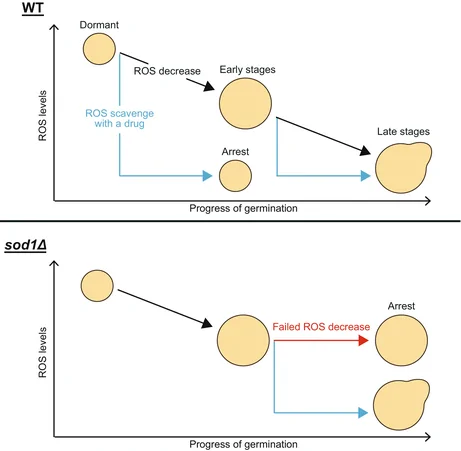

## 1 Introduction

Eukaryotic cells proliferate when sufficient nutrients are available in the environment. When exposed to external stresses such as starvation, however, cells exit the mitotic cycle and suppress their metabolism to enter dormancy. When environmental conditions improve, dormant cells sense them, resume metabolic processes, and restart the division cycle. Dormancy breaking is commonly observed in diverse organisms, such as plant seed germination (reviewed in Nautiyal et al. 2023), yeast spore germination (in Sakai et al. 2025), mammalian oocyte activation (in Telfer et al. 2023), and the reactivation of dormant cancer cells (in Miller et al. 2021). However, the underlying molecular mechanisms remain unclear. In the fission yeast *Schizosaccharomyces pombe*, entry into and exit from spore dormancy, namely sporulation and germination, can be manually controlled by medium exchange. Therefore, analyses of *S. pombe* dormancy breaking are expected to contribute to a general understanding of the molecular mechanisms underlying germination.

*S. pombe* enters a dormant state under nitrogen starvation through sexual differentiation: cellular conjugation, meiosis and sporulation (reviewed in Yamamoto et al. 1997). When carbon sources such as glucose or sucrose are supplied, spores break dormancy and germinate (Hatanaka and Shimoda 2001; Johnke and Padilla 1979; Shimoda 1980). In spores supplied with glucose, activation of the cAMP/PKA signaling pathway induces germination (Hatanaka and Shimoda 2001). In germinating spores, phenomena such as cytoplasmic streaming, morphological changes, and changes in histone H3 levels occur sequentially (Hatanaka and Shimoda 2001; Sakai et al. 2024, 2025; Tsuyuzaki et al. 2020, 2021).

Single‐cell‐based gene expression analyses were previously performed on spores induced to germinate, and differentially expressed genes (DEG) during the germination procedure were identified (Tsuyuzaki et al. 2020). These genes may be potential candidates for dormancy breaking in spores. Gene Ontology (GO) enrichment analyses identified that the DEGs frequently belonged to the following terms: ascospore formation, nucleobase‐containing small molecule metabolic process, detoxification, mRNA metabolic process, and lipid metabolic process.

In this study, we focused on the GO term detoxification as a possible regulator of *S. pombe* spore germination. Genes annotated with the GO term detoxification generally function to neutralize or reduce toxic substances in cells. As the mRNA levels of these genes significantly changed at the initial stage of germination, these factors may be potential candidates that promote germination by modulating the levels of cellular toxic substances. In this study, we focused on reactive oxygen species (ROS) as toxic substances that can be involved in germination.

ROS collectively refer to oxidants such as the superoxide anion radical (O_2_^•−^) and hydrogen peroxide (H_2_O_2_). Intracellular ROS are produced in the mitochondria and plasma membrane by NADPH oxidase, which affects cellular phenomena by oxidizing biomolecules such as nucleic acids, proteins, and lipids. It has also functioned as a modulator of signal transduction under physiological conditions (Lennicke and Cochemé 2021).

Our focus on ROS and detoxification stems primarily from the findings of our single‐cell RNA‐seq analysis. Additionally, the connection between ROS and germination in other organisms further justifies this focus. In plants, ROS promotes seed germination. Imbibed seeds produce ROS, which induces the catabolism of the dormancy hormone abscisic acid and the biosynthesis of the germination hormone gibberellin (Amir et al. 2026; Liu et al. 2010). In addition, both nitric oxide and ROS are required for spore germination of the wheat stripe rust fungus *Puccinia striiformis Westend* f. sp. *tritici* (Yin et al. 2016).

In contrast, in insects, ROS have been reported to contribute to the induction and maintenance of dormancy. For example, cotton bollworm *Helicoverpa armigera* diapausing pupae accumulate ROS in the brain compared to non‐diapausing pupae, which induces lifespan extension by triggering metabolic suppression (Zhang et al. 2022, 2017). Cyclic fluctuations in intracellular ROS regulate the mode of energy metabolism during dormancy (Chen et al. 2021). While these findings indicate that ROS play a significant role in the induction and maintenance of dormancy in insects, it remains unclear whether ROS are involved in the process of dormancy breaking.

Thus, although ROS have been shown to be involved in dormancy, the effects of ROS on dormancy maintenance and breaking vary in each case. Therefore, in this study, we aimed to investigate whether the accumulation or detoxification of ROS influences spore germination in *S. pombe*.

## 2 Results

### 2.1 Optimal Amount of ROS Is Required for Spore Germination

As germination progresses, *S. pombe* spores undergo morphological changes, initially swelling, followed by outgrowth, characterized by the formation of protrusions (Hatanaka and Shimoda 2001). As shown in Figure 1A–C, wild‐type (WT) spores swelled after germination induction and formed protrusions 5 h after induction. The effects of drug‐induced changes in intracellular ROS levels on germination were evaluated. The ROS donor hydrogen peroxide (H_2_O_2_) or the ROS scavenger *N*‐acetylcysteine (NAC) was introduced at the initiation of spore germination, and the germination process was subsequently monitored over time. Both H_2_O_2_ and NAC treatments demonstrated a concentration‐dependent inhibition of the morphological alterations (Figure 1A–C).

**FIGURE 1:**
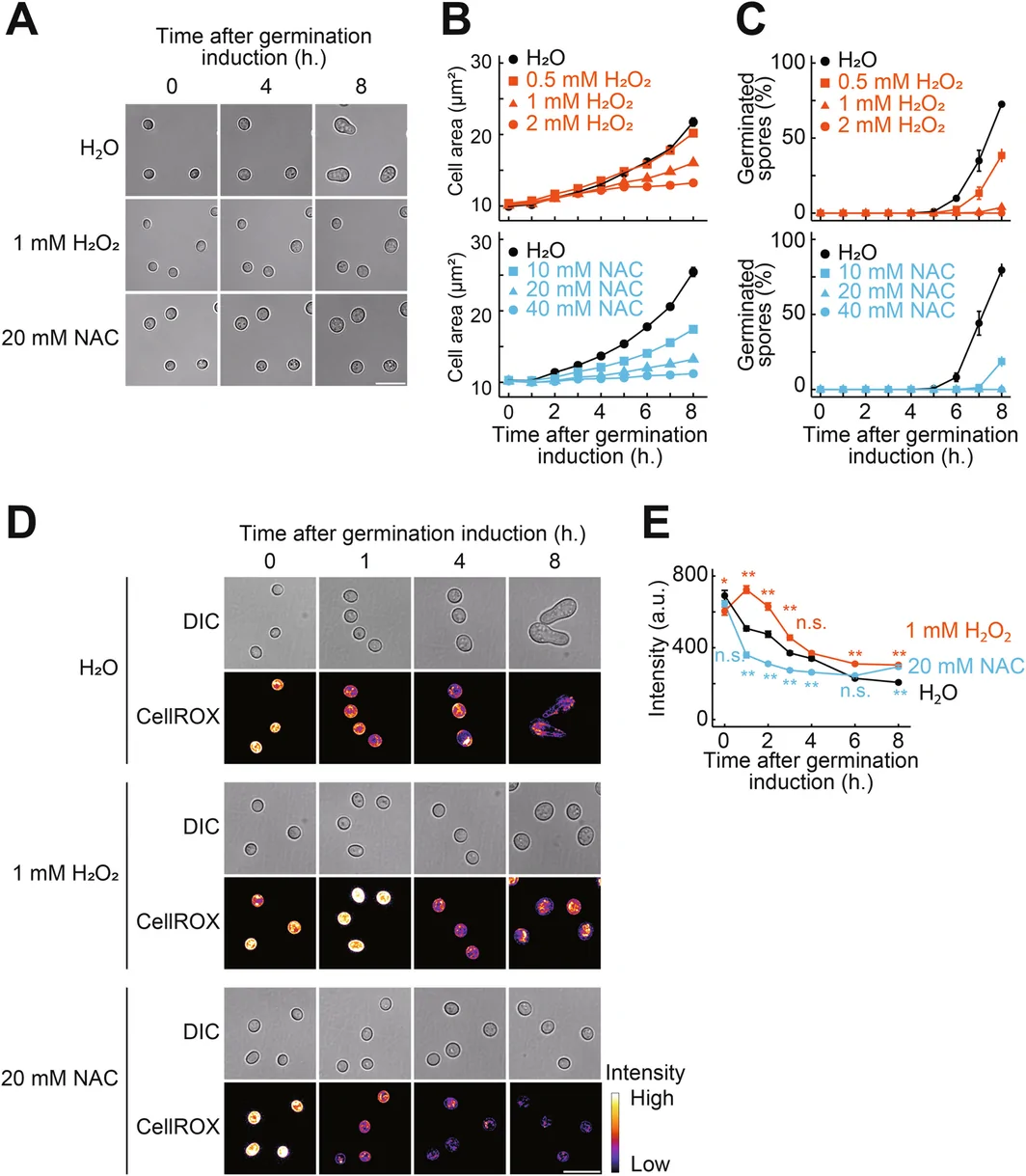
Germination of *S. pombe* spores is inhibited by both hydrogen peroxide and *N*‐acetylcysteine. (A–C) Spores were observed at the indicated time (0–8 h) after 1 mM hydrogen peroxide (H_2_O_2_) or 20 mM *N*‐acetylcysteine (NAC) was added to the media at the onset of germination induction. (A) Spores for each condition at the indicated time points. Scale bar = 10 μm. (B) The area of each spore at each time point was quantified. The results for spores in the presence of varying concentrations of H_2_O_2_ are depicted in red (top), whereas those for spores with NAC are shown in blue (bottom). The values for the control spores (H_2_O) are shown in black in both graphs. Mean ± SE. *N* = 3 independent experiments. *n* ≥ 70 cells. (C) Percentages of spores displaying germination protrusions at each time point after nourishment with drug treatment are shown as in B. The measurement results for spores to which each concentration of H_2_O_2_ or NAC was added at the start of the observation are shown in red (top graph) or blue (bottom), respectively. Mean ± SE. *N* = 3 independent experiments. *n* ≥ 78 cells. (D, E) Spores pre‐stained with the ROS indicator CellROX were observed at the indicated time points after germination induction with either 1 mM H_2_O_2_ or 20 mM NAC. (D) Pseudocolours correspond to the fluorescence intensity of CellROX (a.u.). The relationship between CellROX intensity and color is shown on the far right of the heatmap. Scale bar = 10 μm. (E) CellROX fluorescence intensity (a.u.) of spores observed in D was measured. Mean ± SE. *N* = 3 independent experiments. *n* ≥ 78 cells for each group. Intensities for spores with H_2_O (control; black), spores with 1 mM H_2_O_2_ (red), and spores with 20 mM NAC (blue) are shown. Statistical analyses were performed at each time point using one‐way ANOVA followed by Dunnett's post hoc test. **p < 0.01; *p < 0.05; n.s., *p* > 0.05. Spores with H_2_O (control; black), spores with 1 mM H_2_O_2_ (red), and spores with 20 mM NAC (blue) are shown.

These findings indicate that alterations in intracellular ROS levels, whether increased or decreased due to external influences, inhibit the morphological changes associated with spore germination. The spores exposed to these reagents were subsequently examined to determine whether they retained viability. Spores cultured for 8 h in the presence of H_2_O_2_ or NAC during germination induction were inoculated onto YE5S medium without reagents, and colony formation was measured. No significant difference in colony‐forming ability was observed between H_2_O_2_‐ or NAC‐treated spores and non‐treated spores (Figure S1A), demonstrating that neither reagent affected spore viability.

As these results suggested that the progression of spore germination is sensitive to the amount of intracellular ROS, we monitored the kinetics of intracellular oxidant‐sensitive fluorescence using CellROX (CellROX Green) during the germination process. Note that fluorescence by ROS probes including CellROX in general provides an indicator of intracellular probe oxidation, but it does not identify the subcellular site at which ROS are generated.

In the absence of reagents, the CellROX fluorescence decreased after induction of germination (Figure 1D,E), indicating that the intracellular oxidant‐sensitive signal was high in dormant spores and declined as germination progressed. On the other hand, in spores treated with H_2_O_2_ at germination induction, the CellROX fluorescence, indicating ROS, increased immediately after H_2_O_2_ addition and subsequently decreased (Figure 1D,E). Furthermore, in spores treated with NAC at germination induction, the CellROX signal showed a greater decrease than in spores of the control group (Figure 1D,E). Therefore, ROS levels decreased immediately after the addition of NAC. Taken together, these results show that the intracellular CellROX signal, which was high in dormant spores, decreased as germination progressed. In addition, these results suggest that systems may exist that control ROS at optimal levels throughout the progression of germination.

### 2.2 Artificial Control of the ROS Level Upon Germination Suppressed Fluctuation of Hht1

As the artificial alteration of intracellular ROS levels affected germination in terms of morphological changes in spores, we next examined whether this alteration also influenced intracellular events. A remarkable phenomenon that occurs in the early stages of germination is the reduction of Hht1, which is encoded by one of the histone H3 genes in fission yeast, followed by a rebound of Hht1 in the late stage of germination (Tsuyuzaki et al. 2020). When spores expressing Hht1–GFP were induced to germinate during time‐lapse microscopy, Hht1–GFP fluorescence reportedly decreased from 1 to 6 h after germination induction and then rebounded at 8 h (H_2_O; Figure 2A,B). In spores treated with H_2_O_2_, the initial decrease in Hht1–GFP fluorescence occurred similarly to that in the control spores, but the subsequent increase was not observed (1 mM H_2_O_2_). In contrast, in spores treated with NAC, the initial decrease in Hht1–GFP fluorescence during germination occurred more gradually (20 mM NAC).

**FIGURE 2:**
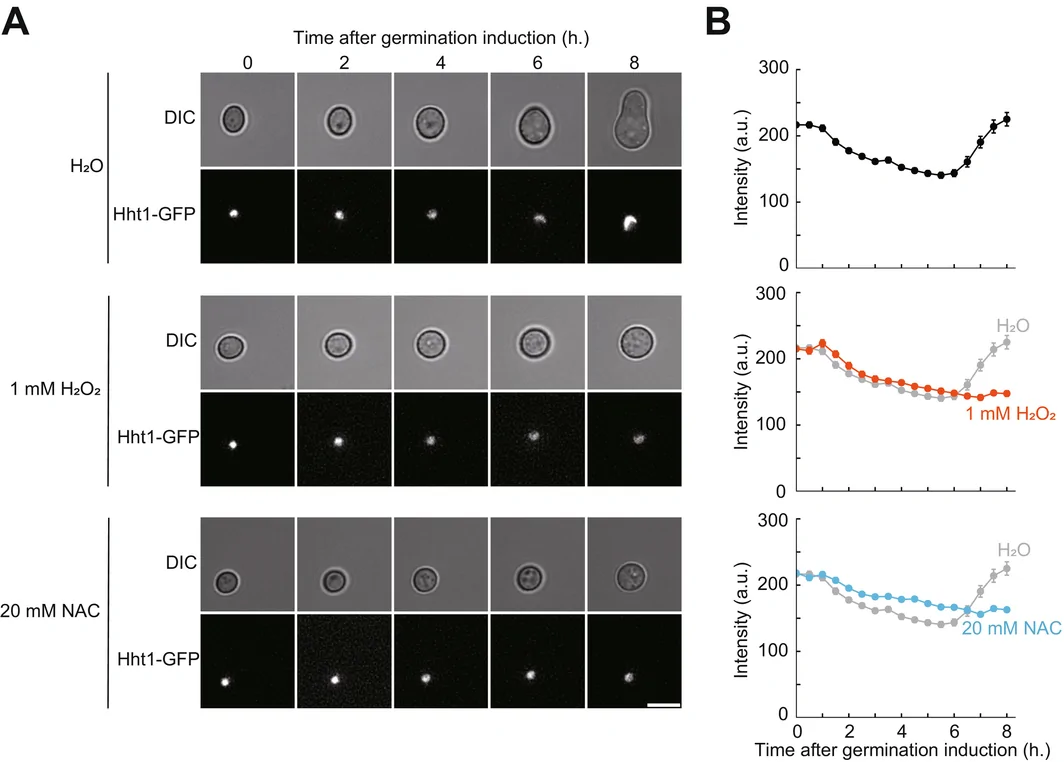
Temporal fluctuation of the Hht1 protein amount upon germination induction is cancelled by H_2_O_2_ or NAC. (A) Germination was induced in spores expressing Hht1–GFP, and time‐lapse imaging was conducted. H_2_O for control, and 1 mM H_2_O_2_ or 20 mM NAC was added upon germination induction. Scale bar = 5 μm. (B) Fluorescence intensity (a.u.) of Hht1–GFP in spore nuclei was measured along the timeline. Mean ± SE. *N* = 3 independent experiments. *n* ≥ 66 cells for each group. Spores with H_2_O for control (black/gray), spores with 1 mM H_2_O_2_ (red), and spores with 20 mM NAC (blue) are shown.

Thus, the artificial reduction of ROS inhibited the decrease in Hht1–GFP, whereas the artificial increase in ROS did not affect the Hht1–GFP decrease in early stages, although it inhibited the upregulation of Hht1–GFP in late stages. These findings indicate that enforced changes in ROS levels affect fluctuations in Hht1 levels during the early and late stages of germination.

### 2.3 Cytoplasmic Fluidisation Upon Germination Was Suppressed by Removal of Intracellular ROS

To further assess the effects of changes in intracellular ROS levels on physiological processes during early stages of germination, we focused on the fluidization of the cytoplasm, which occurs upon dormancy breaking (Figure 3A). The fluidity of the cytoplasm can be monitored by live‐cell imaging of 40 nm‐GEM, genetically encoded multimeric nanoparticles with a 40‐nm diameter through self‐assembly of monomer peptides (Delarue et al. 2018; Sakai et al. 2024). Spores expressing 40 nm‐GEM were induced to germinate and observed every hour for 40 nm‐GEM. As previously reported, cytoplasmic 40 nm‐GEM nanoparticles in dormant WT spores exhibited almost no motility, whereas they actively moved after induction of germination (Figure 3B,C; Movie S1). When NAC was added upon germination induction, the motility of 40 nm‐GEM nanoparticles was severely suppressed. However, the suppression was relatively minor when H_2_O_2_ was added (Figure 3B,C; Movie S1). Thus, the initial fluidization of the cytoplasm upon dormancy breaking was inhibited, particularly when the intracellular ROS level was reduced by external factors.

**FIGURE 3:**
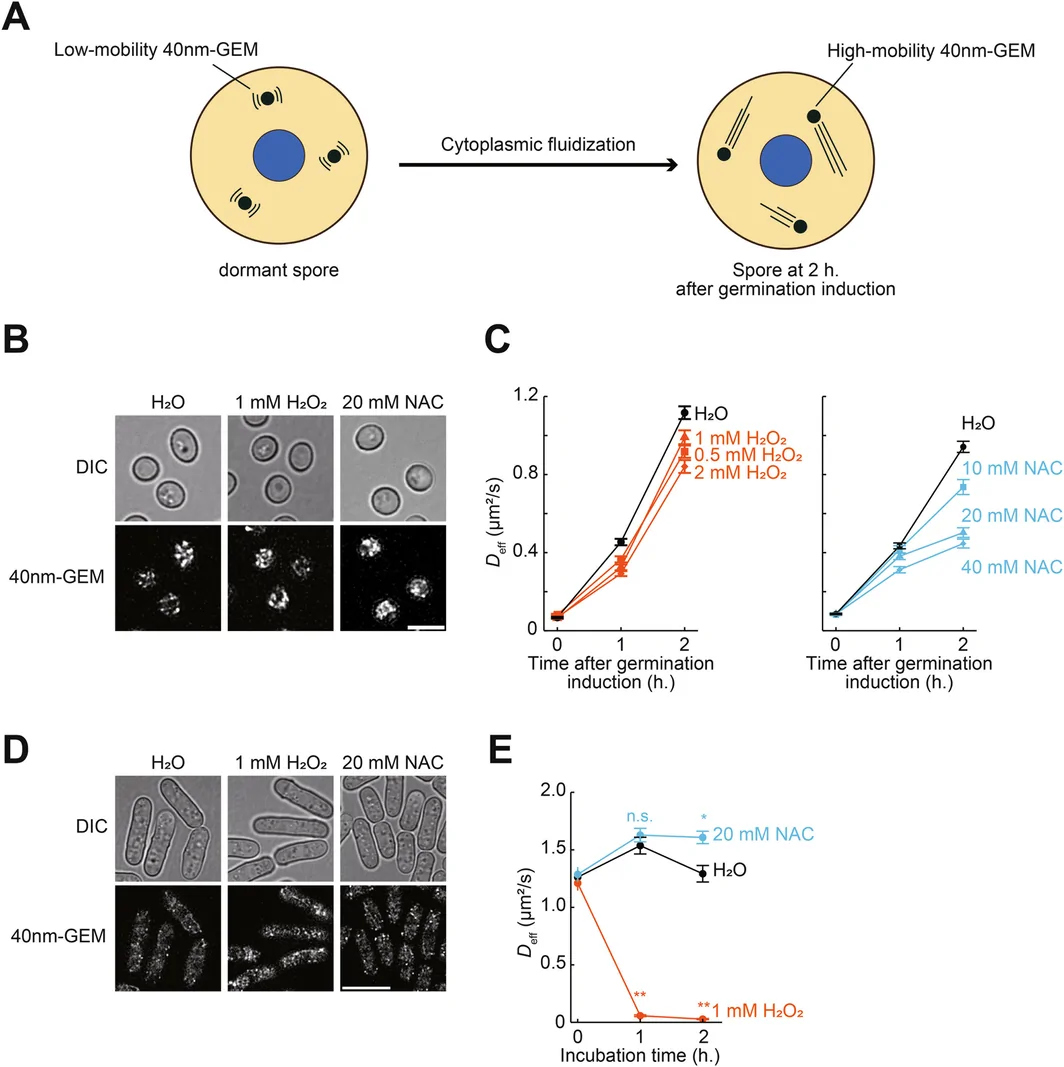
H_2_O_2_ reduces cytoplasmic fluidity, while NAC suppresses the increase in cytoplasmic fluidity during germination. (A) Schematic diagram showing that the motility of 40 nm‐GEM nanoparticles in spores increases upon dormancy breaking. The motility of 40 nm‐GEM in dormant spores is low (left), and that in germinating spores is high (right). (B, C) Spores expressing 40 nm‐GEM were induced to germinate in the absence (H_2_O) or presence of 1 mM H_2_O_2_ or 20 mM NAC and observed at 0–2 h. (B) Representative spores 2 h after germination induction. Scale bar = 5 μm. (C) The effective diffusion coefficient *D*_eff_ (μm^2^/s) of the spores was measured at the indicated time. Mean ± SE. *N* = 3 independent experiments. *n* ≥ 191 cells for each group. (D, E) Vegetative cells expressing 40 nm‐GEM particles treated with 1 mM H_2_O_2_ or 20 mM NAC were observed at 0–2 h. (D) Representative vegetative cells expressing 40 nm‐GEM at 2 h after addition. Scale bar = 10 μm. (E) The effective diffusion coefficient *D*_eff_ (μm^2^/s) of vegetative cells was measured at the indicated time. Mean ± SE. *N* = 3 independent experiments. *n* ≥ 137 cells. Statistical analysis was performed using one‐way ANOVA at each time point, followed by Dunnett's post hoc test. **p < 0.01; *p < 0.05; n.s., *p* > 0.05. For the control spores with H_2_O (black), spores with 1 mM H_2_O_2_ (red), and spores with 20 mM NAC (blue).

In contrast, when H_2_O_2_ was added to vegetative cells, the motility of 40 nm‐GEM nanoparticles sharply decreased, whereas the motility slightly increased in cells treated with NAC (Figure 3D,E; Movie S2). H_2_O_2_ may be toxic, particularly to vegetative cells. Although it is challenging to directly compare ROS levels between spores and vegetative cells due to technical limitations with fluorescent probes, it is notable that dormant spores and those in early germination exhibit relatively high ROS levels compared to the decreased levels in later stages. Importantly, the artificial removal of intracellular ROS suppressed cytoplasmic fluidity in germinating spores but not in vegetative cells, indicating that NAC may inhibit processes specific to spore germination located upstream of cytoplasmic fluidization rather than directly inhibiting the motility of 40 nm‐GEM.

### 2.4 Sod1 Is Required for Progression of Late Stages of Germination

In WT spores, intracellular ROS levels decreased as germination progressed (Figure 1D,E), suggesting that a system for degrading intracellular ROS may operate in germinating spores. Copper‐zinc superoxide dismutase (Sod1) is a major candidate that functions at the most upstream of the known antioxidant system and converts the superoxide anion O_2_^•−^, a type of ROS, into H_2_O_2_. It has been reported that spores of mutants defective in Sod1 activity do not form protrusions (Plante et al. 2017), demonstrating the involvement of Sod1 in germination.

To further investigate the involvement of Sod1 during germination, the ROS state was first monitored in germinating spores of *sod1*Δ pre‐stained with CellROX, and the CellROX fluorescence decreased immediately after germination induction until 4 h, similar to WT spores (Figure 4A,B). However, at 6–8 h after germination induction, CellROX fluorescence remained elevated in *sod1*Δ spores, whereas it continued to decrease in WT spores (Figure 4A,B). These results suggest that Sod1 contributes to the reduction of the ROS signal during the late stages of germination.

**FIGURE 4:**
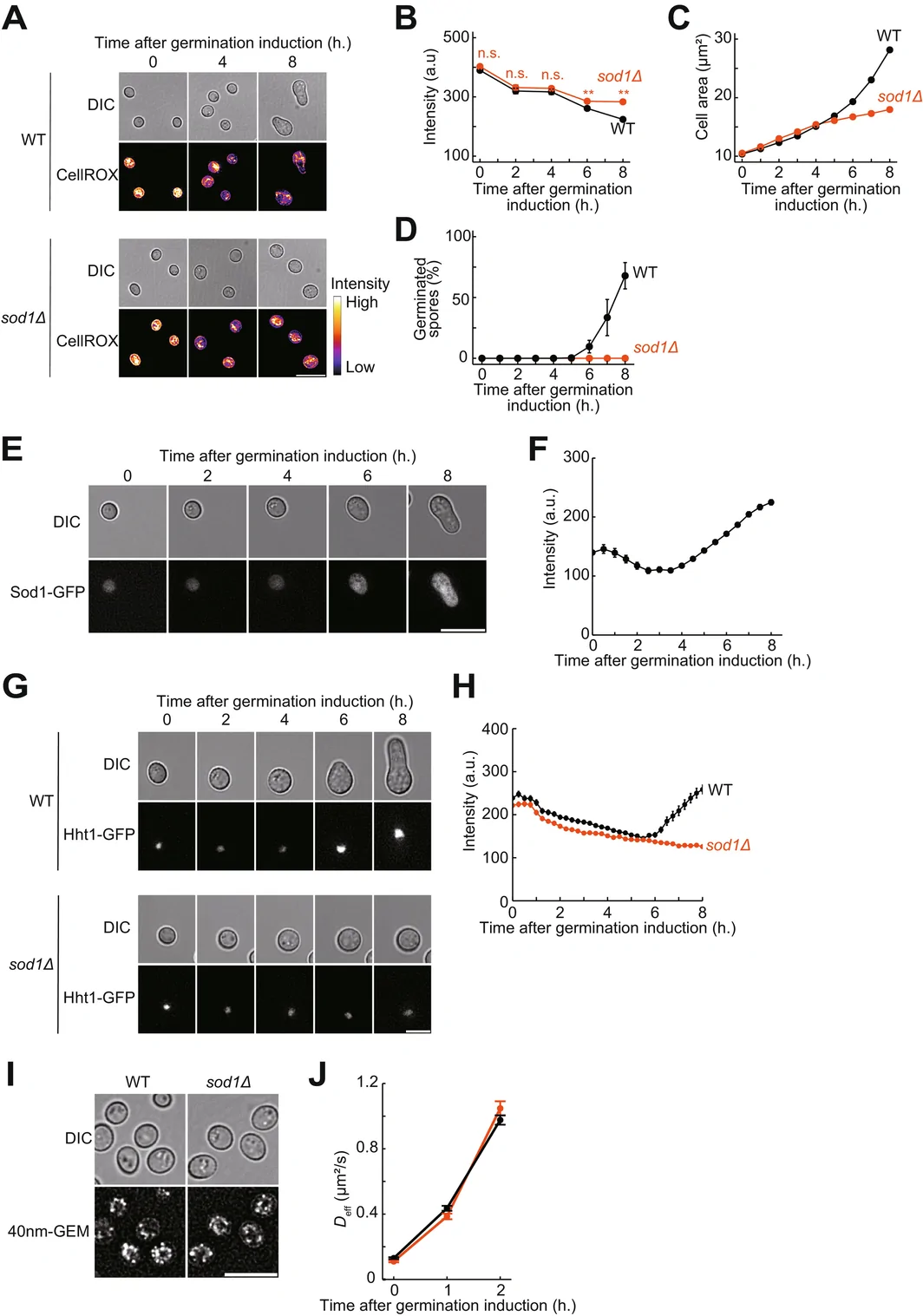
The superoxide dismutase Sod1 promotes cellular projection in the late stage of germination. (A, B) Germination was induced for WT and *sod1*Δ spores pre‐stained with CellROX followed by time‐lapse observation. (A) Pseudo‐colors correspond to the fluorescence intensity of CellROX (a.u.), as shown in the heatmap. Scale bar = 10 μm. (B) The fluorescence intensity of CellROX (a.u.) in each WT (black) and *sod1*∆ (red) spore was quantified. Mean ± SE. *N* = 4 independent experiments. *n* ≥ 160 cells. Statistical analysis was performed using the Welch *t*‐test at each time point. **p < 0.01; n.s., *p* > 0.05. (C) Temporal changes in cellular area during germination. Germination was induced for WT (black) and *sod1*Δ (red) spores and observed using time‐lapse microscopy. Mean ± SE. *N* = 3 independent experiments. *n* ≥ 126 cells. (D) Germination was induced in WT (black) and *sod1*Δ (red) spores as in C. Percentages of spores displaying projections at the indicated time are shown. Mean ± SE. *N* = 3 independent experiments. *n* ≥ 37 cells. (E) Expression of Sod1–GFP during spore germination induced at 0 h. Scale bar = 10 μm. (F) Fluorescence intensity (a.u.) of Sod1–GFP in E was quantified. Mean ± SE. *N* = 3 independent experiments. *n* = 66 cells. (G, H) Germination of WT (black) and *sod1*Δ (red) spores expressing Hht1–GFP was induced for time‐lapse imaging (G). Scale bar = 5 μm. The fluorescence intensity (a.u.) of Hht1–GFP in each spore was quantified (H). Mean ± SE. *N* = 3 independent experiments. *n* ≥ 77 cells. (I, J) Germination of WT (black) and *sod1*Δ (red) spores expressing 40 nm‐GEM particles was induced to monitor their motility at the indicated time. (I) Representative images for 40 nm‐GEM in spores 2 h after induction of germination. Scale bar = 5 μm. (J) The effective diffusion coefficient *D*_eff_ (μm^2^/s) of the spores was quantified at each time point. Mean ± SE. *N* = 3 independent experiments. *n* ≥ 290 cells. Statistical analysis was performed using one‐way ANOVA. No significant difference was observed between WT and *sod1*Δ spores.

Observation of morphological changes upon germination of *sod1*Δ spores showed that the germination projection was never formed, as previously reported (Plante et al. 2017). However, in the early stages, up to 4 h after germination induction, *sod1*Δ spores swelled to a degree comparable to that of WT spores (Figure 4C,D). Since swelling of *sod1*Δ spores was comparable to that of WT spores, this suggests that *sod1*Δ spores underwent normal morphological changes up to the stage preceding projection formation. Time‐lapse imaging of Sod1–GFP upon dormancy breaking revealed that the expression of Sod1–GFP increased after 6–8 h of germination induction. Together, these results indicate that Sod1 contributes to the reduction of the ROS signal during late‐stage germination, after Sod1–GFP abundance increases, whereas its influence in earlier stages remains minor. However, the present data do not determine the subcellular compartment in which Sod1 acts on ROS.

We examined whether Sod1 also affects the temporal kinetics of Hht1 levels. *sod1*Δ spores exhibited a decrease in Hht1–GFP fluorescence in early stages of germination, as in WT spores, whereas the rebound in later stages did not occur (Figure 4G,H). To further test whether Sod1 is involved in early stages of germination, cytoplasmic fluidization upon germination induction was monitored using 40 nm‐GEM. The motility of 40 nm‐GEM in *sod1*Δ spores increased to a degree comparable to that in WT spores (Figure 4I,J). Thus, Sod1 was dispensable for cytoplasmic fluidization during early germination. These findings, combined with the persistent CellROX signal observed in *sod1*Δ spores and the elevated expression of Sod1–GFP in the later stages of wild‐type spores, indicate that Sod1 plays a role in regulating intracellular redox homeostasis during late stages of germination.

### 2.5 Sod1 Facilitates Germination by Detoxifying ROS During Later Stages

To investigate whether the defects in protrusion formation in *sod1*Δ spores were due to the accumulation of intracellular ROS, particularly in late stages, NAC was added to CellROX‐stained WT and *sod1*Δ spores 4 h after induction to counteract ROS accumulation in the late stages (Figure 5A–C). In WT spores, as shown above (Figure 1A–C), NAC treatment at the time of germination induction inhibited germination projection. In contrast, adding NAC at 4 h had only a minor effect, as intracellular ROS had already been degraded by that time (WT; Figure 5A–C). In *sod1*Δ spores, the addition of NAC at 4 h reduced ROS and also led to a partial recovery of germination protrusion (*sod1*∆; Figure 5A–C). Thus, counteracting ROS accumulation in *sod1*Δ spores during late stages of germination restored their ability to form protrusions. In line with this, the defects of *sod1*Δ cells in the rebound of Hht1–GFP in late stages of germination (Figure 4G,H) were partially suppressed when NAC was added 4 h after germination induction (Figure 5D,E). We collectively concluded that the breakdown of intracellular ROS by Sod1 facilitates the advancement of germination in its later stages.

**FIGURE 5:**
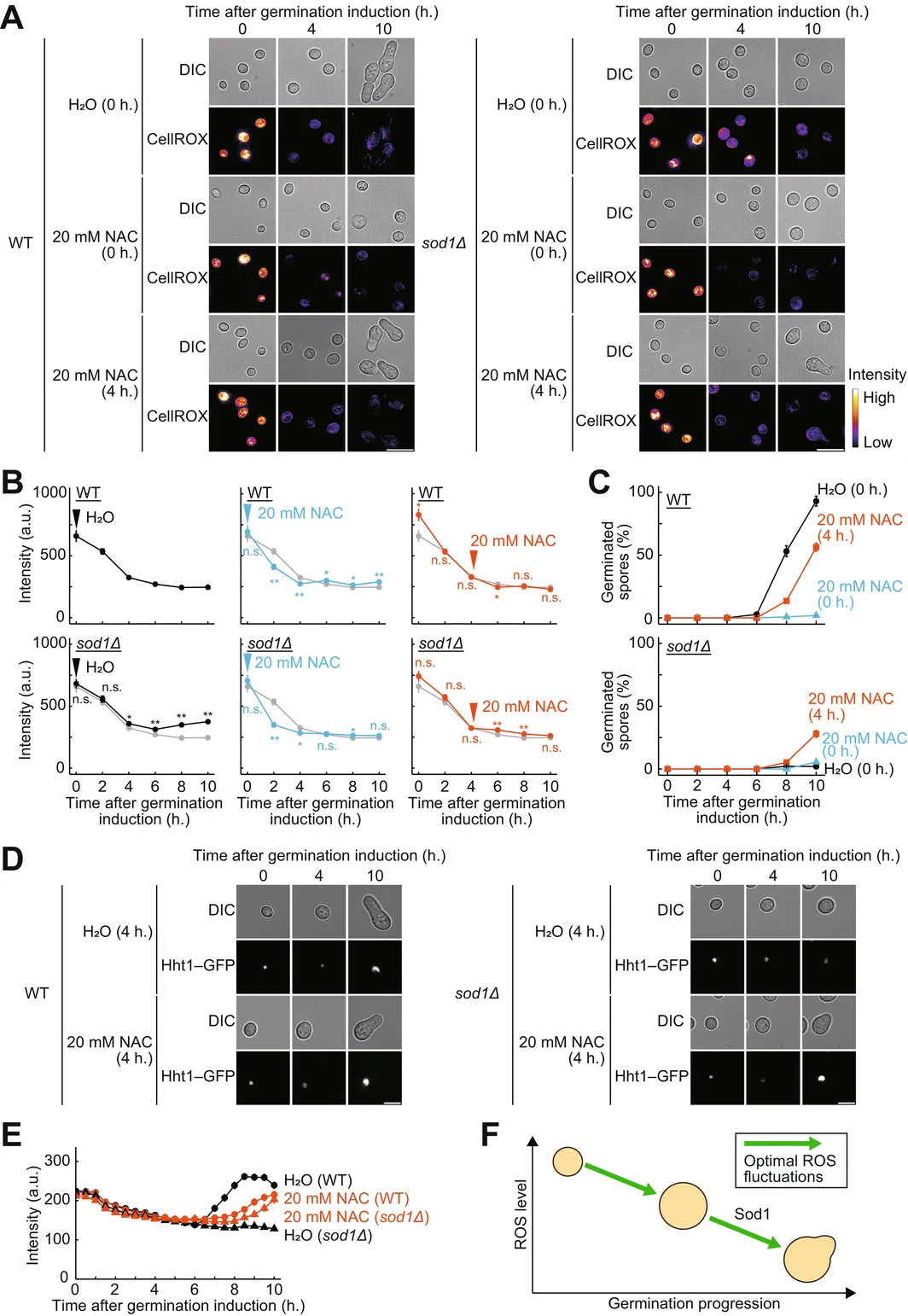
Intracellular ROS accumulated in the absence of Sod1 inhibits progression of spore germination in late stages. (A–C) Germination of WT and *sod1*∆ spores pre‐stained with CellROX was induced. 20 mM NAC was added to media upon germination induction (0 h, blue) or at a later stage (4 h, red). For control, H_2_O was added at 0 h (black/gray). (A) Pseudo‐colors indicate fluorescence intensities (a.u.) of CellROX, as shown in the heatmap. Scale bar = 10 μm. (B) CellROX fluorescence intensity (a.u.) was quantified at each time point. Arrowheads indicate the time at which NAC or H_2_O was added. Black lines in the top left and bottom left indicate WT and *sod1*∆ spores treated with H_2_O (control), respectively. Gray lines in the other graphs indicate the reprise of WT spores treated with H_2_O (control) for comparison. Mean ± SE. *N* = 3 independent experiments. *n* ≥ 43 cells. Statistical analysis was performed at each time point using Welch's *t*‐test between samples with NAC and H_2_O. **p < 0.01; *p < 0.05; n.s., *p* > 0.05. (C) Percentages of spores displaying germination projections at each time point. Mean ± SE. *N* = 3 independent experiments. *n* ≥ 28 cells. (D, E) Expression of Hht1–GFP in WT and *sod1*Δ spores to which NAC or H_2_O (for control) was added 4 h after germination induction. Scale bar = 5 μm. The fluorescence intensity (a.u.) of Hht1–GFP was quantified (E). Fluorescence intensity is shown for WT spores to which H_2_O (black circles) or 20 mM NAC (red circles) was added 4 h after germination induction, as well as for *sod1*Δ spores to which H_2_O (black triangles) or 20 mM NAC was added (red triangles) at 4 h. The arrowhead indicates the time point at which 20 mM NAC was added. Mean ± SE. *N* = 3 independent experiments. *n* ≥ 79 cells. (F) A schematic for the stepwise reduction of intracellular ROS levels during *S. pombe* germination. The second reduction in ROS is facilitated by Sod1 and promotes late‐stage germination events, such as projection formation.

## 3 Discussion

In this study, we showed that proper regulation of intracellular ROS levels is required for normal progression of spore germination in fission yeast, and that the optimal amount of ROS may decrease as germination progresses. Relatively high levels of ROS exist in dormant spores, and these levels decrease as germination proceeds from the initial stage (Figure 1D,E). Additionally, we demonstrated that the antioxidant function of Sod1 is necessary for the reduction of intracellular ROS, which is then required for proper progression of germination events in late stages, such as protrusion formation and increase of the histone H3 protein Hht1.

In contrast, an important limitation of the present study is that CellROX fluorescence does not define the subcellular site of ROS production. Although the fluorescence signal provides evidence for changes in intracellular probe oxidation during dormancy and germination, it cannot distinguish contributions from the cytoplasm, mitochondria, endoplasmic reticulum, peroxisomes, or other intracellular compartments. Future analyses using compartment‐targeted redox sensors and organelle markers will be required to determine the source and spatial distribution of ROS during dormancy breaking and germination.

Despite the limitation, the relatively high intracellular ROS signal probed by CellROX observed in dormant spores may reflect redox changes that occur during spore formation and/or prolonged nutrient limitation. The source of ROS in dormant *S. pombe* spores and the physiological conditions that establish this redox state remain to be determined. In natural environments, spores may be exposed to multiple stress conditions, including nutrient depletion, oxidative stress, temperature fluctuation, and other environmental challenges (Ohtsuka et al. 2022). These conditions could influence ROS production or ROS‐scavenging capacity before germination (Papadakis and Workman 2015; Pekmez et al. 2008; Roux et al. 2006). ROS production can also be influenced by mitochondrial metabolism (Zuin et al. 2008).

Although the molecular events of germination have been extensively investigated in *Saccharomyces cerevisiae* , intracellular ROS dynamics during spore germination have not been characterized. As *S. cerevisiae* spores retain functional mitochondria and undergo marked metabolic and cytoplasmic remodeling upon germination induced by nourishment (Joseph‐Strauss et al. 2007), it will be informative to determine whether the temporal ROS regulation identified here is conserved in budding yeast spores.

In vegetatively growing *S. pombe* cells, intracellular ROS levels are influenced by metabolic state and oxidative stress. Elevated ROS production has been reported under replication stress, inappropriate cell‐cycle progression (Marchetti et al. 2006), mitochondrial dysfunction (Zuin et al. 2008), and exposure to oxidants (Papp et al. 2012). By contrast, whether basal ROS levels show a temporal change during an unperturbed vegetative cell cycle remains unclear. Thus, the chronological ROS dynamics observed during spore germination may represent a distinct redox‐regulatory feature associated with the transition from dormancy to proliferative growth.

Spore germination and G0 recovery both involve re‐entry into proliferative growth after a non‐proliferative state. During nitrogen starvation‐induced G0, wild‐type *S. pombe* cells exhibit weak ROS signals, whereas the proteasome mutant *mts3‐1* shows pronounced ROS accumulation. This suggests that ROS levels are maintained low during G0 dormancy (Takeda et al. 2010), although the temporal dynamics of ROS during recovery from G0 dormancy remain to be characterized. In contrast, in spores, ROS levels are higher before dormancy breaking and are reduced upon dormancy breaking, suggesting that distinct ROS control mechanisms may operate in G0 cells and in spores. Nonetheless, maintaining ROS homeostasis is crucial for both the maintenance of the G0 state and spore germination.

Throughout this study, NAC was used to remove intracellular ROS, and the addition of NAC to WT spores at the initial stage of the germination procedure inhibited early events such as swelling, followed by protrusion formation, a drop in the Hht1 level, and cytoplasmic fluidization. In contrast, the addition of NAC to WT spores at a late stage (4 h) caused no specific defects in late events, such as protrusion formation and a rebound of Hht1. This demonstrates that intracellular ROS are essential for dormancy breaking, particularly in the initial stage of the germination procedure, but not in late stages.

On the contrary, Intracellular ROS has another impact on the progression of germination in late stages. An artificial increase in ROS in late stages of germination, achieved by the knockout of superoxide dismutase Sod1, inhibited the overall late events. These results led us to postulate a relationship between intracellular ROS and germination progression (Figure 5F). Intracellular ROS levels need to be high in spores at the initial stage of dormancy breaking, whereas ROS needs to be detoxified by Sod1 in the subsequent stages for the progression of germination.

Sod1–GFP accumulated in the nucleus during the later stages of germination, despite the CellROX signal being mostly in the cytoplasm. In vegetative budding yeast cells, Sod1 has been shown to translocate to the nucleus in response to oxidative stress, where it contributes to genome stability and transcriptional regulation (Tsang et al. 2014). In fission yeast, Sod1 may be retained predominantly outside the nucleus during dormancy and early germination, or its nuclear import may be limited by the low‐fluidity cytoplasmic state. Moreover, although the CellROX signal at this stage appeared largely extranuclear, this probe does not necessarily identify the subcellular source of ROS. These factors may account for the observed discrepancy between the nuclear accumulation of Sod1 and the cytoplasmic CellROX signal.

In WT spores, ROS levels decreased as germination progressed. In *sod1*Δ spores, ROS levels remained high even in late stages, and late germination events failed to occur. This was partially suppressed by the addition of NAC in the late stage of germination. For instance, compared to spores undergoing normal germination, protrusion formation and increase in Hht1 were delayed and did not reach complete recovery (Figure 5A–E). It is possible that the artificial reduction of ROS by NAC addition did not fully reproduce the physiological fluctuations in ROS levels that decrease during germination. The reduction in CellROX fluorescence during late stages indicated that not only O_2_^•–^ but also overall ROS, including H_2_O_2_, decreased. As Sod1 is an enzyme that converts O_2_^•–^ to H_2_O_2_, multiple redox enzymes other than Sod1, such as catalase Ctt1 and glutathione peroxidase Gpx1, which function downstream of the Sod1 pathway, may cooperatively regulate ROS levels during late stages of germination.

The cytoplasm fluidises upon germination, which is one of the earliest events of the germination procedure as far as recognized (Sakai et al. 2024). Fluidization was affected when intracellular ROS levels were artificially altered. First, the addition of H_2_O_2_ to spores partially inhibited fluidization. The inhibitory effect on fluidization was also observed when H_2_O_2_ was added to vegetative cells, suggesting that H_2_O_2_ generally reduces cytoplasmic fluidity, irrespective of cellular conditions. It remains unclear whether H_2_O_2_ inhibits cytoplasmic fluidization directly or indirectly. Excessive oxidative stress caused by H_2_O_2_ may affect intracellular conditions, such as the cellular metabolic state and intracellular pH, thereby potentially reducing fluidity (Munder et al. 2016; Parry et al. 2014). The addition of NAC during nourishment inhibited cytoplasmic fluidization at dormancy breaking to a greater extent than the addition of H_2_O_2_, although the effect of NAC was not observed when added to vegetative cells. This indicates that NAC may not directly affect fluidization; rather, it may indirectly influence upstream events specific to breaking the dormancy of spores to induce cytoplasmic fluidization.

In any case, an adequate amount of intracellular ROS is required for cytoplasmic fluidization at dormancy breaking. Thus, altering the amount of ROS affected both intracellular conditions and the morphological characteristics of the outer shape, indicating that ROS influences dormancy breaking in multiple ways.

The impact of ROS on dormancy breaking in eukaryotes appears to be common, but remains controversial. It has recently been reported that ROS function as modulators of signal transduction and play important roles in physiological processes (Lennicke and Cochemé 2021), indicating a common requirement for ROS control in developmental cues, such as dormancy breaking. In plant seed germination, an increase in ROS in early stages promotes the progression of germination by inducing both the degradation of the dormancy hormone abscisic acid and the biosynthesis of the germination hormone gibberellin (Amir et al. 2026; Liu et al. 2010). In addition, both nitric oxide and ROS are required for spore germination in the wheat stripe rust fungus *Puccinia striiformis* Westend f. sp. *tritici* (Yin et al. 2016). During dormancy in the flesh fly *Sarcophaga crassipalpis* , intracellular ROS levels exhibit periodic fluctuations that regulate metabolic arousal (Chen et al. 2021). Thus, ROS dynamically modulates metabolic activity throughout the dormancy period rather than remaining static, although the relationship between ROS fluctuation and dormancy breaking remains unknown.

This study revealed that the temporal fluctuation of ROS in *S. pombe* involves a certain level of ROS in the initial stages, followed by a stepwise reduction in ROS during germination. The first reduction in ROS occurs immediately after the initiation of germination. This seems to be influenced by unknown factors other than Sod1, as initial ROS reduction was observed even in *sod1*∆ spores. Subsequently, in the later stages of germination, Sod1‐dependent ROS reduction occurs, which is necessary for various events involved in dormancy breaking, possibly through the modulation of metabolic enzymes and/or gene expression systems. Understanding the mechanisms by which ROS are required at the very initial stage of germination and how ROS are reduced as germination proceeds will advance our knowledge of dormancy breaking.

## 4 Experimental Procedures

### 4.1 Yeast Strains, Media, and Genetics

The *S. pombe* strains used in this study are listed in Table 1. This study used standard methods for *S. pombe* (Moreno et al. 1991). For vegetative growth of *S. pombe*, YE5S (0.5% yeast extract, 3% d‐glucose, and five supplements [adenine 0.10 g/L, uracil 0.05 g/L, L‐leucine 0.05 g/L, l‐lysine 0.05 g/L, l‐histidine 0.10 g/L]) was used. The same five‐supplement mixture was used in Edinburgh minimal media: EMM + C + N + 5S and EMM + N + 5S. To induce sporulation, cells cultured on YE5S agar plates were resuspended in a mixture of leucine (5 mg/mL) and uracil (1.25 mg/mL) solution, spotted onto sporulation agar (SPA) plates, and incubated at 26.5°C for 3 days. The methods for inducing germination are described below.

**TABLE 1:** Fission yeast strains used in this study.

Strain name	Genotype	Source
TH683	*h90*	This study
TH586	*h90 hht1–GFP–bsd*	This study
TH764	*h90 2L::Padh21–40nm‐GEM<<nat*	Sakai et al. 2024
TH551	*h90 sod1::kan*	This study
TH820	*h90 hht1–GFP–bsd sod1::kan*	This study
TH537	*h90 sod1–GFP–kan*	This study

### 4.2 Spore Purification

The spots on SPA plates contain spores, asci and other types of cells including mitotic or meiotic ones. To selectively collect spores, the aqueous two‐phase system (ATPS) was used (Imada 2025) as follows. Cells spotted on SPA plates were resuspended in ddH_2_O and centrifuged at 376 × *g* for 3 min to remove the supernatant. A mixture of 40% (w/v) polyethylene glycol, TE buffer [pH 7.5; 10 mM Tris–HCl, 1 mM EDTA] and 0.1 M lithium acetate [pH 7.5], was mixed with 1 M sodium sulphate at a 1:3 volumetric ratio and added to the cell pellet and vortexed (Imada 2025). The cell suspension was left at room temperature for 3 min to allow the spore cells to separate into the upper layer, which was then transferred to a new 1.5 mL tube, and ddH_2_O was added. The mixture was centrifuged at 376 × *g* for 3 min to remove the supernatant. The cell pellet was resuspended in EMM containing a nitrogen source (0.5% NH_4_Cl) and the appropriate supplements.

### 4.3 Assays for Colony‐Forming Ability

After purification, the spores were cultured in EMM supplemented with carbon and nitrogen sources and 5 supplements (EMM + C + N + 5S) medium containing 1 mM H_2_O_2_ or 20 mM NAC at 30°C for 8 h. After incubation, approximately 200 spore cells were counted using a hemocytometer, collected, and plated onto YE5S plates to induce germination followed by colony formation. The number of colonies formed was counted after incubation at 30°C for 3 days. For the 200 colonies plated on the plate, the number of colonies formed on the plate was counted, and the colony forming efficiency (%) was calculated as their ratio.

### 4.4 Detection of ROS

Purified spores (1 mL, concentration: 1 × 10^6^ cells/mL) obtained using the ATPS method were incubated with 5 μM CellROX Green (Thermo Fisher Scientific) at 30°C for 2 h. After incubation, the spores were washed 3 times with EMM + N + 5S medium (lacking carbon sources) and observed.

### 4.5 Microscopy

Standard microscopy procedures were performed as previously described (Sato et al. 2009). The observation system consisted of a DeltaVision‐SoftWorx image acquisition system (Applied Precision) equipped with an inverted microscope (IX71 or IX81) and a CoolSNAP HQ2 CCD camera (Photometrics). Spores were purified, stained with CellROX if necessary, resuspended in EMM + N + 5S lacking carbon sources, and placed on a glass‐bottom dish (Matsunami‐glass) pre‐coated with lectin derived from *Glycine max* (Sigma). The same medium was used to fill the dish prior to observation. To induce spore germination, EMM + N + 5S containing 40% glucose (20× concentration) was added.

Observations of spore morphology, Hht1–GFP, and Sod1–GFP were performed at 30°C. Observations were conducted every 15 min for a total of 10.5 h, during which germination was induced 30 min after the start of the observation.

For CellROX imaging, cells were kept in the incubator at 30°C until the next time point and then placed under a microscope for snapshots at each time point. Images were acquired as 10 sections along the *Z*‐axis at 0.4 μm intervals.

For the observation of 40 nm‐GEM, image acquisition was conducted at 0.2‐s intervals for a total of 6 s, and this was repeated multiple times at each time point. Images were acquired as single sections along the *Z*‐axis. For the observation of vegetative cells, the cells were cultured overnight at 30°C in SD + 5S medium. Cells in culture (concentration: 0.5–1.0 × 10^7^ cells/mL) were collected and washed with EMM + C + N + 5S. The observations were performed in the same manner as described above.

### 4.6 Image Analyses

Observation images acquired along the *Z*‐axis were deconvolved using the SoftWoRx software (v3.7.0 and v6.5.1) and then projected into a single image using the sum projection algorithm of the software. The cell area and fluorescence intensity were quantified using Fiji (Schindelin et al. 2012). For quantification of the fluorescence intensity of Hht1–GFP, the average fluorescence intensity within the nuclear region exhibiting fluorescence was measured, and the average fluorescence intensity of the extracellular region was subtracted as background. For quantification of the fluorescence intensity of CellROX, the average fluorescence intensity across the entire spore region was measured, and the average fluorescence intensity of the extracellular region was subtracted as background.

### 4.7 Analyses of 40 nm‐GEM Nanoparticles

Cells expressing 40 nm‐GEM (Sakai et al. 2024) were observed for 6 s at 0.2‐s intervals. As the initial 1.0 s right after the start of observation were not recorded exactly at 0.2‐s intervals, images from 1.0 to 6.0 s were used for analyses. To measure the mobility of each particle, the Particle Tracker 2D/3D function in the Fiji plugin Mosaic was used with the following parameters: radius = 3, cutoff = 0, per/abs = flexible, and Brownian dynamics, as previously described (Sakai et al. 2024). The quantified data were used to calculate the effective diffusion coefficient (*D*_eff_).

## Author Contributions

**Tatsuya Hasegawa:** data curation, formal analysis, investigation, methodology, resources, validation, visualization, writing – original draft, funding acquisition. **Masamitsu Sato:** conceptualization, project administration, supervision, validation, writing – review and editing, funding acquisition.

## Funding

This study was supported by JST SPRING (Grant number B2R101263201) to T.H. This study was also supported by JSPS KAKENHI (Grant numbers 18K19347, 21H00261, 23K27173, 25H02582 and 26K02009), Ohsumi Frontier Science Foundation, Institute for Fermentation, Osaka, and Waseda University grants for Special Research Projects Grant Numbers 2018B‐222, 2019C‐570, 2020R‐038, 2021C‐227, 2022C‐164, 2023C‐167, 2023R‐026 and 2024C‐490 to M.S.

## Ethics Statement

The authors have nothing to report.

## Conflicts of Interest

The authors declare no conflicts of interest.

## Supporting information


**Figure S1:** Neither H_2_O_2_ nor NAC affects colony formation ability of *S. pombe* spore (A) WT spores were cultured for 8 h in liquid EMM media containing 1 mM H_2_O_2_, 20 mM NAC, or H_2_O (control), and germination was induced. For each group, 200 cells were counted under a microscope and plated onto YE5S plates. The number of colonies formed after 3 days of incubation at 30°C was counted, and the percentage relative to the 200 plated cells was calculated. Mean ± SE. *N* = 3 independent experiments. Statistical analysis was performed using one‐way ANOVA followed by Dunnett's post hoc test, comparing the control, H_2_O_2_, and NAC samples. n.s., *p* > 0.05.
**Movie S1:** The mobility of 40 nm‐GEM in germinating spores. Spores expressing 40 nm‐GEM were induced to germinate in the absence (H_2_O) or presence of 1 mM H_2_O_2_ or 20 mM NAC and observed at 0–2 h. Images were recorded at 0.2‐s intervals, and this was repeated multiple times at each time point. Those images were acquired as a single section along the *Z*‐axis. The video shows representative spores at 2 h after germination induction. The video playback speed is shown as 10 frames per second, and the video timestamps are given in min:s. Scale = 5 μm.
**Movie S2:** The mobility of 40 nm‐GEM in vegetative cells. Vegetative cells expressing 40 nm‐GEM were treated with H_2_O, 1 mM H_2_O_2_ or 20 mM NAC and observed at 0–2 h. Images were recorded at 0.2‐s intervals. Those images were acquired as a single section along the *Z*‐axis. The video shows representative vegetative cells at 2 h after addition. The video playback speed is shown as 10 frames per second, and the video timestamps are given in min:s. Scale = 10 μm.

## Data Availability

The data that support the findings of this study are available from the corresponding author upon reasonable request.
