# Supplementary material for: ROS Is Necessary for Dormancy Breaking in Fission Yeast Spores, While Its Downregulation by Sod1 Is Crucial for Later Events
Source: Genes Cells. 2026 Nov 3;31(6):e70157. doi: 10.1111/gtc.70157 (PMC13728812; doi:10.1111/gtc.70157)
Supplement: Supplementary file 1 [file GTC-31-0-s001.pdf]

Supporting Information

ROS is necessary for dormancy breaking in fission yeast spores, while its downregulation by Sod1 is crucial for later events

Tatsuya Hasegawa & Masamitsu Sato

Supplementary figure S1
Supplementary movies S1 and S2

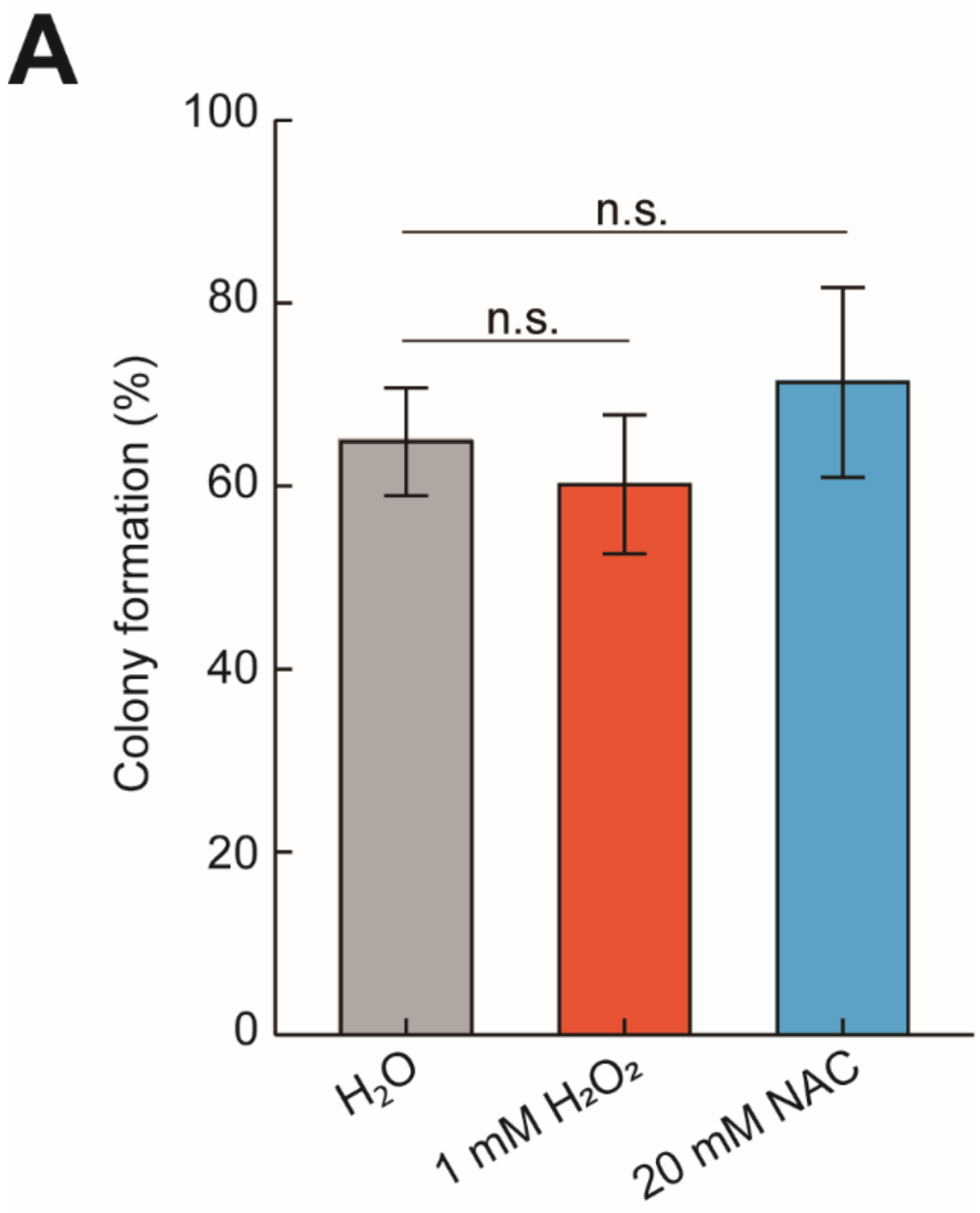

Figure S1 Neither H_2O_2 nor NAC affects colony formation ability of *S. pombe* spores

(A) WT spores were cultured for 8 h in liquid EMM media containing 1 mM H_2O_2, 20 mM NAC, or H_2O (control), and germination was induced. For each group, 200 cells were counted under a microscope and plated onto YE5S plates. The number of colonies formed after 3 days of incubation at 30°C was counted, and the percentage relative to the 200 plated cells was calculated. Mean ± SE. N = 3 independent experiments. Statistical analysis was performed using one-way ANOVA followed by Dunnett's post hoc test, comparing the control, H_2O_2, and NAC samples. n.s., $p > 0.05$.

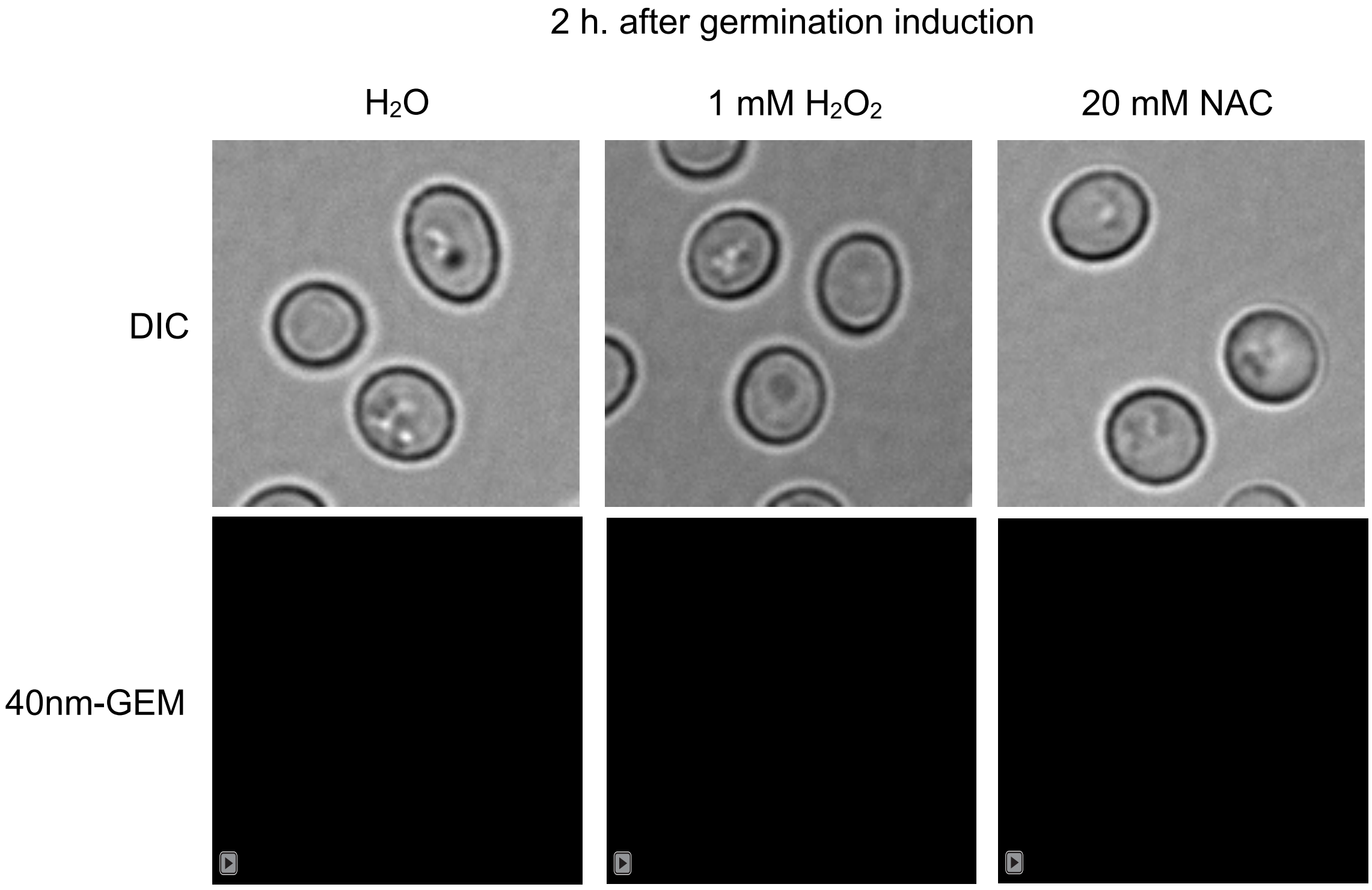

Movie S1 The mobility of 40nm-GEM in germinating spores

Spores expressing 40nm-GEM were induced to germinate in the absence (H_2O) or presence of 1 mM H_2O_2 or 20 mM NAC and observed at 0–2 h. Images were recorded at 0.2-s intervals, and this was repeated multiple times at each time point. Those images were acquired as a single section along the Z-axis. The video shows representative spores at 2 h after germination induction. The video playback speed is shown as 10 frames per second, and the video timestamps are given in min:s. Scale = 5 μm.

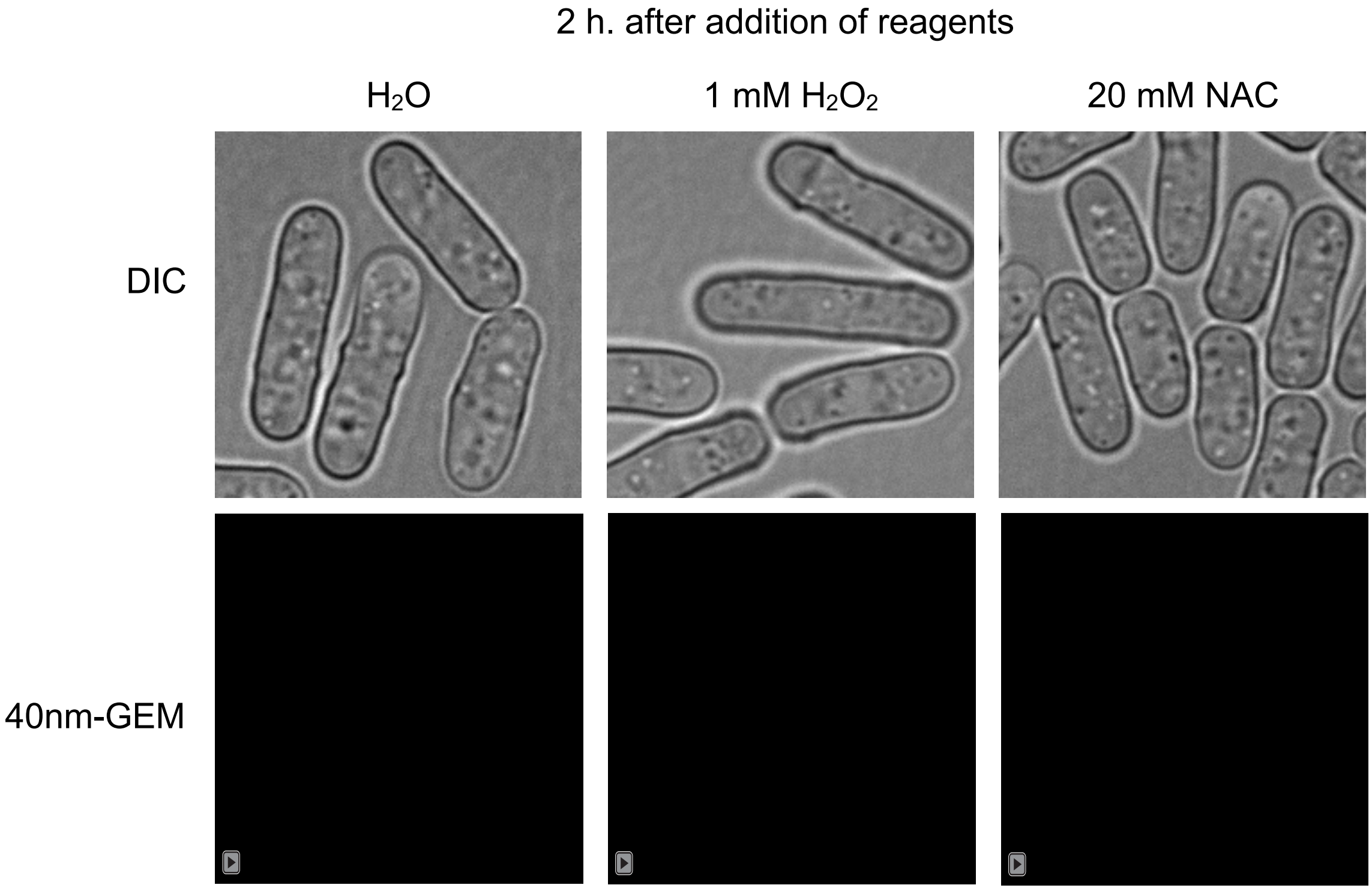

Movie S2 The mobility of 40nm-GEM in vegetative cells

Vegetative cells expressing 40nm-GEM were treated with H_2O, 1 mM H_2O_2 or 20 mM NAC and observed at 0–2 h. Images were recorded at 0.2-s intervals. Those images were acquired as a single section along the Z-axis. The video shows representative vegetative cells at 2 h after addition. The video playback speed is shown as 10 frames per second, and the video timestamps are given in min:s. Scale = 10 μm.
